# Polymorphisms in the tumor necrosis factor gene and susceptibility to Behcet’s disease: An updated meta-analysis

**Published:** 2013-09-12

**Authors:** Min Zhang, Wang-Dong Xu, Peng-Fei Wen, Yan Liang, Jie Liu, Hai-Feng Pan, Dong-Qing Ye

**Affiliations:** 1 Department of Epidemiology and Biostatistics, School of Public Health, Anhui Medical University, Hefei, Anhui, PR China; 2 Anhui Provincial Laboratory of Population Health & Major Disease Screening and Diagnosis, Anhui Medical University, Hefei, Anhui, PR China

## Abstract

**Propose:**

Studies investigating the association between the tumor necrosis factor (TNF) gene polymorphisms and Behcet’s disease (BD) report conflicting results. The aim of this meta-analysis was to assess the association between TNF gene polymorphisms and BD.

**Methods:**

A systematic literature search was conducted to identify all relevant studies. Pooled odds ratios (ORs) with 95% confidence intervals (CIs) were used to estimate the strength of the association.

**Results:**

A total of 16 articles, involving 1,708 patients with BD and 1,910 healthy controls, were included in the meta-analysis. Overall, a significant association was found between BD and the TNF −308A/G polymorphism (OR=0.730, 95% CI=0.608–0.877, p=0.001). Meta-analysis of TNF −238A/G showed significant association with BD (OR=1.512, 95% CI=1.155–1.979, p=0.003). The TNF −1031C allele showed significant association with BD (OR=1.549, 95% CI=1.190–2.015, p=0.001). Similarly, the meta-analysis showed a significant association of the TNF −857T/C polymorphism with BD (OR=0.758, 95% CI=0.593–0.968, p=0.027). Stratification by ethnicity revealed that the −308A/G and −857T/C polymorphisms were associated with BD in the Asian group, while the −238A/G and −1031C/T polymorphisms were associated with BD in the Caucasian population.

**Conclusions:**

The results of our meta-analysis suggest that TNF (−308A/G, −238A/G, −1031C/T, and −857T/C) polymorphisms are associated with susceptibility to BD.

## Introduction

Behcet’s disease (BD) is a chronic relapsing inflammatory disease characterized by recurrent oral and genital mucous ulcers and ocular and skin lesions [1]. BD also involves vessels of all sizes, central nervous system disease, and gastrointestinal tract and thrombotic events, which are less frequent but can be life-threatening [1]. Ocular inflammation is often present at the disease onset of BD and is the initial manifestation in approximately 20% of patients. If not present at disease onset, ocular involvement occurs most commonly within 2–4 years, eventually affecting more than 50% of patients [2]. The typical form of ocular involvement is relapsing remitting uveitis that may cause significant damage to the intraocular structures. Much less frequently, ocular involvement may present in the form of conjunctival ulcers, episcleritis, scleritis, or extraocular muscle paralysis due to neurologic involvement [3-5]. Intraocular inflammation may involve the anterior or posterior segment or, more commonly, both. Since lesions affecting the posterior segment are persistent in nature and correlated with significant vision loss, anterior or posterior classification of uveitis is therapeutically and prognostically important [6]. The pathogenesis of BD remains unknown, but evidence has indicated that genetic and immunological mechanisms are related to BD. During the past two decades, the genetic participation in the pathogenesis of BD has been widely investigated. The HLA-B51 locus is recognized as a genetic marker of susceptibility to BD [7,8]. Two recent genome-wide association studies (GWASs) [9,10] indicated associations between single nucleotide polymorphisms (SNPs) of the major histocompatibility complex (MHC) class I region, some cytokines, and BD susceptibility. Studies have also implicated the abnormality of lymphocyte function in patients with BD, especially for T cell subsets. Saadoun et al. demonstrated the promotion of Th17 responses and the suppression of regulatory T cells (Tregs) that were driven by interleukin (IL)-21 production and that correlate with BD activity [11]. In a study of Japanese patients, Th22 cells played an important role in enhancing the inflammatory response in patients with BD who have uveitis through producing large amounts of IL-22 and tumor necrosis factor**-**α (TNF**-**α) [12]. In addition, epidemiological studies found that people genetically originating from an endemic region who emigrated to different nations appear to have a significantly lower risk of BD, such as Japanese living in Hawaii [13] and the mainland United States and Turks living in Germany [14], suggesting that environmental factors may play a role in BD susceptibility. Bacterial and viral infections, as well as abnormal antigen presentation, have been implicated in initiating immunopathological pathways leading to the disease onset of BD, such as *Streptococcus sanguis*, Herpes simplex virus 1, and heat shock proteins 60/65 [15-18]. To date, the most comprehensive immunopathogenesis hypothesis speculates that the etiology of BD can be triggered by environmental factors in genetically susceptible individuals, especially microbiological factors [19].

TNF**-**α, an important proinflammatory cytokine, is secreted primarily by mononuclear phagocytic cells [20]. It is implicated in the pathogenesis of several inflammatory disorders. TNF**-**α is involved in various physiologic and pathologic processes, such as inflammation initiation, immunoregulation, proliferation, and apoptosis [21]. Overexpression of proinflammatory cytokines from various cellular sources seems to be related to the severity of inflammatory responses in BD. Serum levels of TNF-α are increased in patients with active BD as well as secretion of TNF-α from stimulated peripheral blood mononuclear cells [22,23]. Individual differences in TNF-α production are related to several single nucleotide polymorphisms (SNPs) in the TNF gene region [24-26]. Furthermore, monocytes from patients with BD can spontaneously generate large amounts of TNF-α [27]. Yamashita et al. showed that the levels of TNF-β produced by the γδT cells in patients with BD were higher than those of healthy controls [28]. However, treatment with TNF-α inhibitors indicated a dramatic anti-inflammatory effect against major BD lesions, particularly for uveitis [29-31]. These findings indicated that TNF-α might play a pivotal role in the pathogenesis of BD.

The TNF gene is encoded in the class III region of the MHC on chromosome 6p21.3 [32]. Over the last decade, numerous studies have investigated the relationship between TNF gene polymorphisms and BD risk [23,33-47]. However, the results of previous studies are not consistent. The discord may be attributable to small sample size, various racial and ethnic backgrounds, uncorrected multiple hypothesis testing, and publication bias.

Meta-analysis is a statistical method for combining the results of several studies to produce a single estimate of the major effect with enhanced precision. Meta-analysis is considered a powerful tool for pooling inconsistent results from different studies [48]. Touma et al. performed a meta-analysis to assess the association between TNF gene polymorphisms and BD risk, but this meta-analysis included only ten studies [49]. More studies concerning the association between SNPs and BD risk have been reported in recent years [43-47]. Thus, it seems necessary to perform a meta-analysis that includes the most updated data to investigate the relationships between TNF gene polymorphisms and the risk of BD.

## Methods

### Publication search

A systematic literature search in PubMed, Elsevier Science Direct, the China National Knowledge Infrastructure database (CNKI), and the Chinese Biomedical database (CBM) was performed to identify articles. References in the studies were reviewed to find additional studies regarding the association between TNF gene polymorphisms and BD risk. The text words were as follows: “Behcet’s disease or Behcet syndrome” and “tumor necrosis factor or tumor necrosis factor gene” combined with “single nucleotide polymorphism or polymorphism or polymorphisms.” The languages were limited to English and Chinese. The last search was updated on August 1, 2012.

### Inclusion and exclusion criteria

The inclusion criteria were defined as follows: a) The design was a case-control or cohort study; b) the studies evaluated the association between TNF gene polymorphisms (−308A/G, −238A/G, −1031C/T, −857T/C, −863A/C, −376A/G) and BD risk; c) the studies provided sufficient data to calculate the odds ratio (OR); and d) genotype distribution of the control population is in Hardy–Weinberg equilibrium (HWE). Studies were excluded if one of the following existed: a) The studies contained overlapping data, or b) studies included family members who had been studied because of analysis based on linkage considerations.

### Data extraction

Data were collected by two independent investigators (Xu and Wen). The characteristics of the selected articles are shown in Table 1, including first author, year of publication, study population, ethnicity, number of cases and controls, findings about the polymorphisms investigated in these studies, and HWE (p value). The study populations comprised Koreans, Lebanese, Iranians, Moroccans, Tunisians, Turks, and Germans. The Asian subgroup included Korean, Lebanese, and Iranian populations. Moroccan and Tunisian populations were classified in the African subgroup and others in the Caucasian subgroup.

**Table 1:** Characteristics of individual studies included in the meta-analysis

First author	Year	Population	Ethnicity	Case	Control	Genotyping methods	Association		HWE (p value)
							P value (allelic contrast)		
Lee	2003	Korean	Asian	94	94	PCR-SSP	TNF −308A/G	NS	0.667
Duymaz-Tozkir	2003	Turkish	Caucasian	99	96	PCR-RFLP	TNF −308A/G	NS	0.806
							TNF −376A/G	NS	0.793
Ates	2006	Turkish	Caucasian	107	102	PCR	TNF −308A/G	NS	0.254
							TNF −238A/G	NS	0.76
							TNF −376A/G	NS	0.88
Akman	2006	Turkish	Caucasian	99	103	PCR-RFLP	TNF −1031C/T	p=0.018	0.084
Park	2006	Korean	Asian	254	344	PCR-RFLP	TNF −308A/G	p=0.010	0.988
							TNF −238A/G	NS	0.175
							TNF −1031C/T	p=0.030	0.354
							TNF −857T/C	NS	0.456
							TNF −863A/C	p=0.008	0.382
Chang	2007	Korean	Asian	115	114	PCR	TNF −308A/G	NS	0.332
							TNF −238A/G	NS	0.735
							TNF −1031C/T	NS	0.666
							TNF −857T/C	NS	0.284
							TNF −863A/C	NS	0.873
Alayli	2007	Turkish	Caucasian	80	105	PCR-SSP	TNF −238A/G	p=0.001	0.264
Kamoun	2007	Tunisian	African	89	157	PCR-RFLP	TNF −1031C/T	p=0.015	0.99
Storz(1)	2008	German	Caucasian	92	51	PCR	TNF −238A/G	NS	0.599
Storz(2)	2008	Turkish	Caucasian	30	20	PCR	TNF −238A/G	NS	0.814
Akman	2008	Turkish	Caucasian	82	77	PCR	TNF −1031C/T	p=0.023	0.595
Arayssi	2008	Lebanese	Asian	48	90	NA	TNF −308A/G	NS	0.707
							TNF −238A/G	NS	0.701
							TNF −1031C/T	NS	0.068
							TNF −857T/C	NS	0.657
Dilek	2009	Turkish	Caucasian	97	127	PCR-SSP	TNF −308A/G	NS	0.1
Bonyadi	2009	Turkish	Caucasian	53	79	PCR-RFLP	TNF −308A/G	NS	0.277
							TNF −1031C/T	p<0.001	0.909
Ates	2010	Turkish	Caucasian	102	102	ARMS-PCR	TNF −308A/G	NS	0.359
Amirzargar	2010	Iranian	Asian	147	137	PCR-SSP	TNF −308A/G	NS	0.052
Radouane	2012	Moroccan	African	120	112	PCR	TNF −308A/G	NS	0.521
							TNF −238A/G	NS	0.448
							TNF −857T/C	NS	0.355
							TNF −863A/C	NS	0.147
							TNF −376A/G	NS	0.658

NA: not available; NS: not significant, HWE: Hardy–Weinberg equilibrium.

### Statistical analysis

Allele frequencies at the TNF gene polymorphisms from the individual study were determined by the counting method. HWE was tested using the χ^2^ test (significant at the 0.05 level). The strength of association between the gene polymorphisms and BD susceptibility was assessed with ORs and 95% confidence intervals (CIs).

The χ^2^ test-based Q statistic was used to examine the heterogeneity of between-studies [50]. The I^2^ statistic measures the degree of inconsistency in the studies by computing what percentage of the total variation across studies was due to heterogeneity rather than by chance. A high I^2^ value indicated a higher probability of the existence of heterogeneity (I^2^=0% to 25%, no heterogeneity; I^2^=25% to 50%, moderate heterogeneity; I^2^=50% to 75%, large heterogeneity; and I^2^=75% to 100%, extreme heterogeneity). If the p value of the heterogeneity Q statistic was less than 0.10, the random effects model was selected. Otherwise, a fixed-effects model was adopted.

Publication bias was estimated using Egger’s linear regression test and a funnel plot. If the p value was less than 0.05, statistically significant publication bias might exist [51].

All the statistical analyses for the meta-analysis were performed with STATA statistical software (version 11.0 STATA Corp, College Station, TX).

## Results

### Literature search and study characteristics

**Figure 1:**
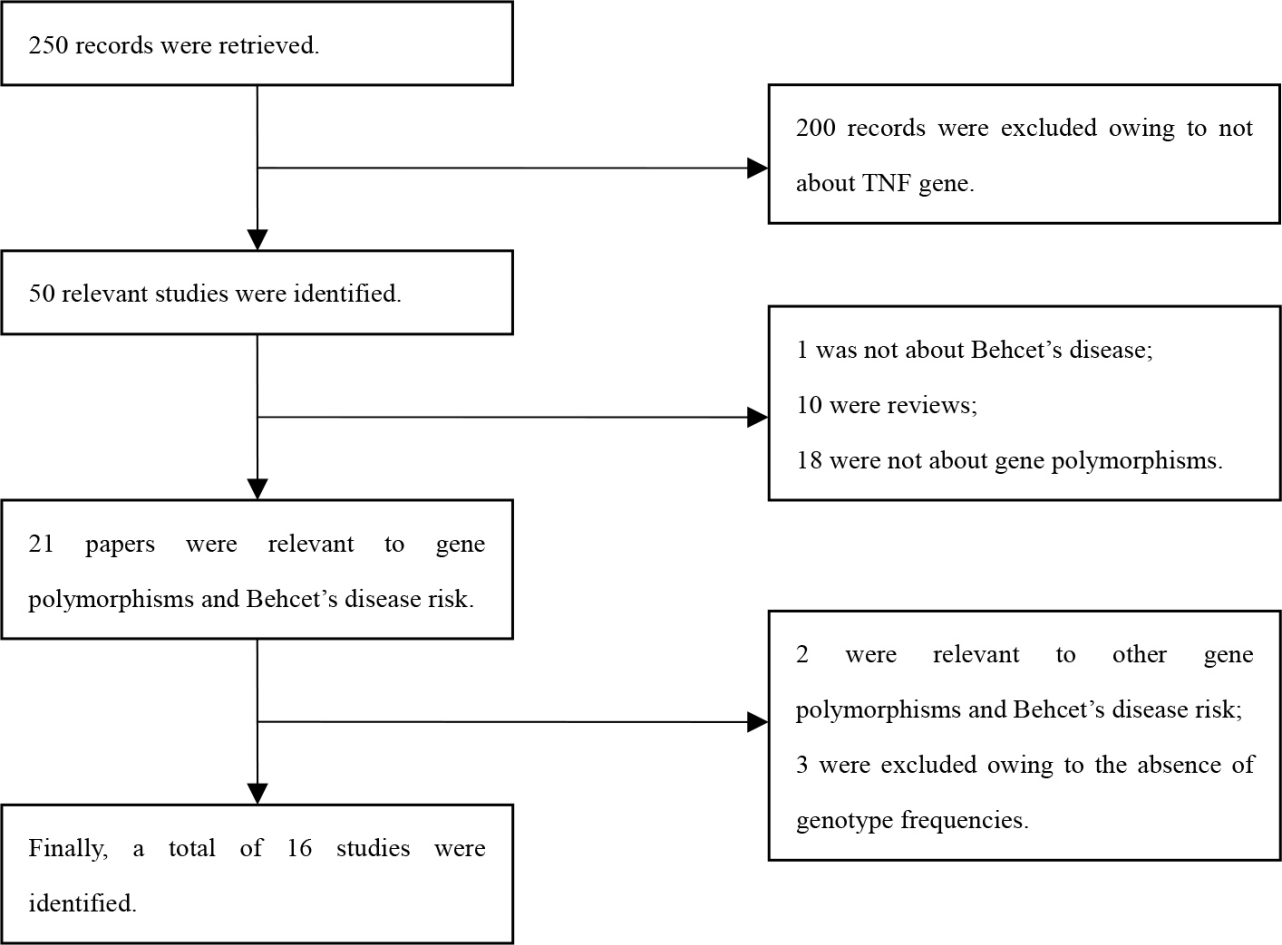
Process for selecting studies.

The process for selecting the studies is shown in Figure 1. Fifty potentially relevant studies were reviewed, and 16 articles met the inclusion criteria and were finally included in our meta-analysis. Of the 16 articles, one study [40] included two cohorts; therefore, each cohort was considered a separate study. Finally, a total of 17 case-control studies in 16 articles were identified [23,33-47], including 1,708 patients with BD and 1,910 healthy controls. There were 11 studies on −308A/G, eight studies on −238A/G, seven studies on −1031C/T, four studies on −857T/C, three studies on −863A/C, and three studies on −376A/G. Nine studies involved Caucasian populations [23,34,35,38,40,41,43-45], five studies involved Asian populations [33,36,37,42,46], and two studies involved African populations [39,47]. The main characteristics of each study included in this meta-analysis are shown in Table 1.

### Meta-analysis of tumor necrosis factor gene polymorphisms in Behcet’s disease

A summary of the meta-analysis of the relationship between TNF gene polymorphisms and BD is listed in Table 2.

**Table 2:** Meta-analysis of the TNF gene polymorphisms in BD

Polymorphisms	Population	Number of studies	Sample size		Test of association				Test of heterogeneity			Egger’s test (P)
			Case control		OR (95%CI)	Z	P	Model	χ*^2^*	P	*I^2^* (%)	
TNF −308A/G	Overall	11	1232	1397	0.730(0.608–0.877)	3.37	0.001	F	13.28	0.208	24.7	0.317
A versus G allele	Asian	5	654	779	0.676(0.511–0.894)	2.75	0.006	F	4.24	0.375	5.7	0.066
	Caucasian	5	458	506	0.833(0.627–1.108)	1.25	0.21	F	7.85	0.11	47	0.565
	African	1	120	112	0.638(0.400–1.017)	1.89	0.059	NA	NA	NA	NA	NA
TNF −238A/G	Overall	8	842	938	1.512(1.155–1.979)	3.01	0.003	F	5.96	0.544	0	0.002
A versus G allele		8^a^	NA	NA	1.521(1.159–1.995)	3.03	NA	NA	NA	NA	NA	NA
	Asian	3	413	548	1.421(0.876–2.303)	1.42	0.154	F	0.66	0.72	0	0.627
	Caucasian	4	309	278	1.556(1.074–2.253)	2.34	0.019	F	5.2	0.158	42.3	0.02
		4^a^	NA	NA	1.574(1.083–2.288)	2.38	NA	NA	NA	NA	NA	NA
	African	1	120	112	1.548(0.790–3.033)	1.27	0.203	NA	NA	NA	NA	NA
TNF −1031C/T	Overall	7	738	964	1.549(1.190–2.015)	3.26	0.001	R	13.54	0.035	55.7	0.89
C versus T allele	Asian	3	415	548	1.203(0.967–1.496)	1.65	0.098	F	2.11	0.348	5.3	0.542
	Caucasian	3	234	259	2.171(1.581–2.981)	4.79	<0.001	F	2.01	0.366	0.6	0.575
	African	1	99	103	1.654(1.098–2.493)	2.41	0.016	NA	NA	NA	NA	NA
TNF −857T/C	Overall	4	533	660	0.758(0.593–0.968)	2.22	0.027	F	0.45	0.93	0	0.949
T versus C allele	Asian	3	326	310	0.757(0.583–0.983)	2.09	0.037	F	0.45	0.799	0	0.974
	African	1	120	112	0.763(0.375–1.553)	0.75	0.456	NA	NA	NA	NA	NA
TNF −863A/C	Overall	3	489	570	1.101(0.707–1.713)	0.43	0.671	R	6.31	0.043	68.3	0.45
A versus C allele	Asian	2	369	458	1.091(0.551–2.158)	0.25	0.803	R	6.15	0.013	83.7	NA
	African	1	120	112	1.082(0.623–1.878)	0.28	0.779	NA	NA	NA	NA	NA
TNF −376A/G	Overall	3	326	310	0.438(0.188–1.024)	1.9	0.057	F	0.24	0.889	0	0.756
A versus G allele	Caucasian	2	206	198	0.476(0.142–1.597)	1.2	0.23	F	0.19	0.66	0	NA
	African	1	120	112	0.405(0.123–1.334)	1.49	0.137	NA	NA	NA	NA	NA

BD: Behcet’s disease, OR: odds ratio, CI: confidence interval, F: fixed effects model, R: random effects model, NA: not available ^a^Adjusted using the ‘‘trim and fill’’ method.

### Tumor necrosis factor −308A/G polymorphism and Behcet’s disease

**Figure 2:**
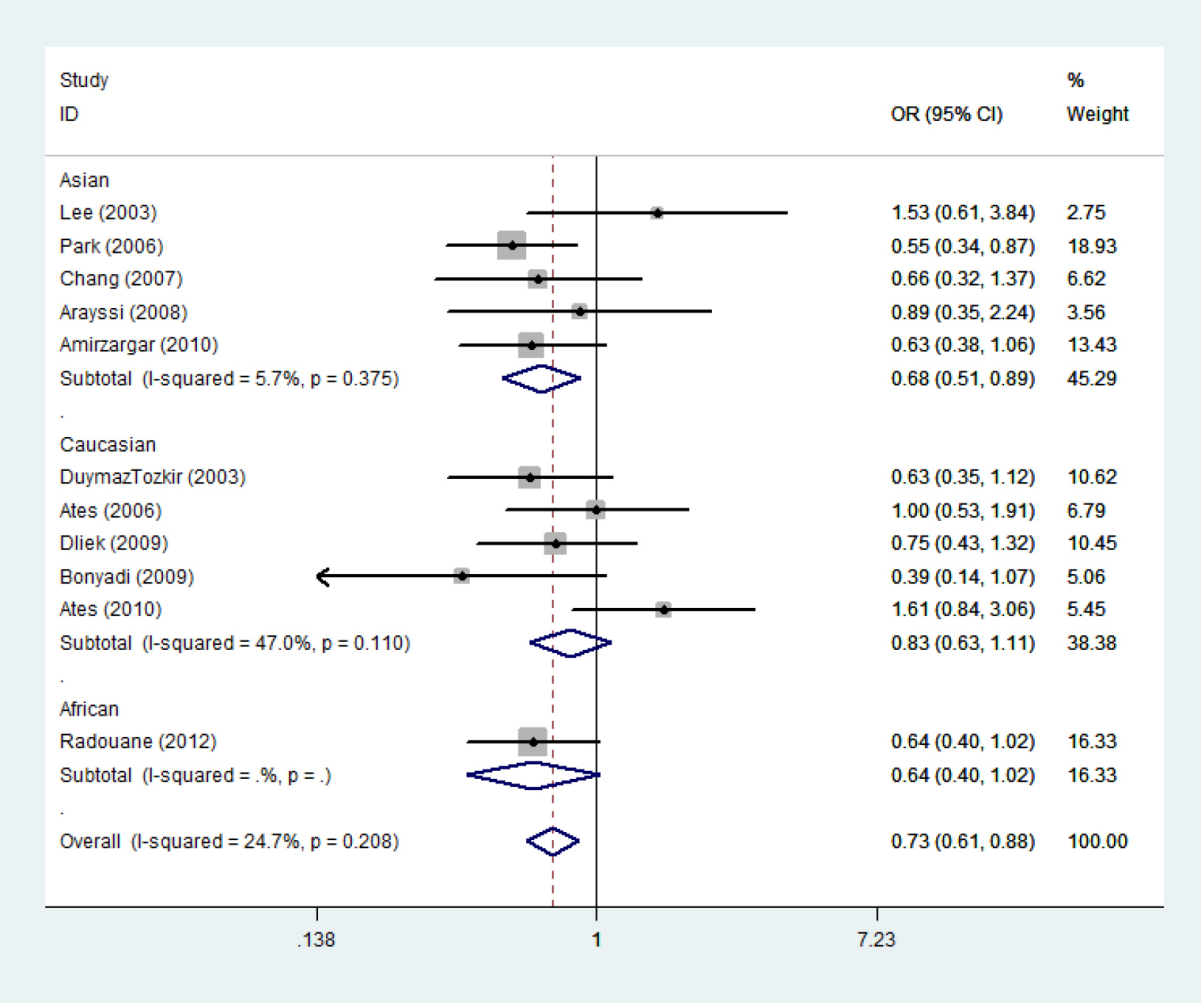
Odds ratios and 95% confidence intervals for individual studies and pooled data for the association between the A versus G allele of the tumor necrosis factor −308A/G polymorphism and Behcet’s disease.

Eleven studies determined the relationship between the −308A/G polymorphism and BD risk [33-37,42-47]. The total sample size for patients with BD and healthy controls was 1,232 and 1,397, respectively. Meta-analysis revealed an association between −308A and BD risk in the overall population (OR=0.730, 95% CI=0.608–0.877, p=0.001; Figure 2). Stratification by ethnicity indicated that the −308A allele was significantly associated with BD risk in the Asian population (OR=0.676, 95% CI=0.511–0.894, p=0.006; Figure 2).

### Tumor necrosis factor −238A/G polymorphism and Behcet’s disease

**Figure 3:**
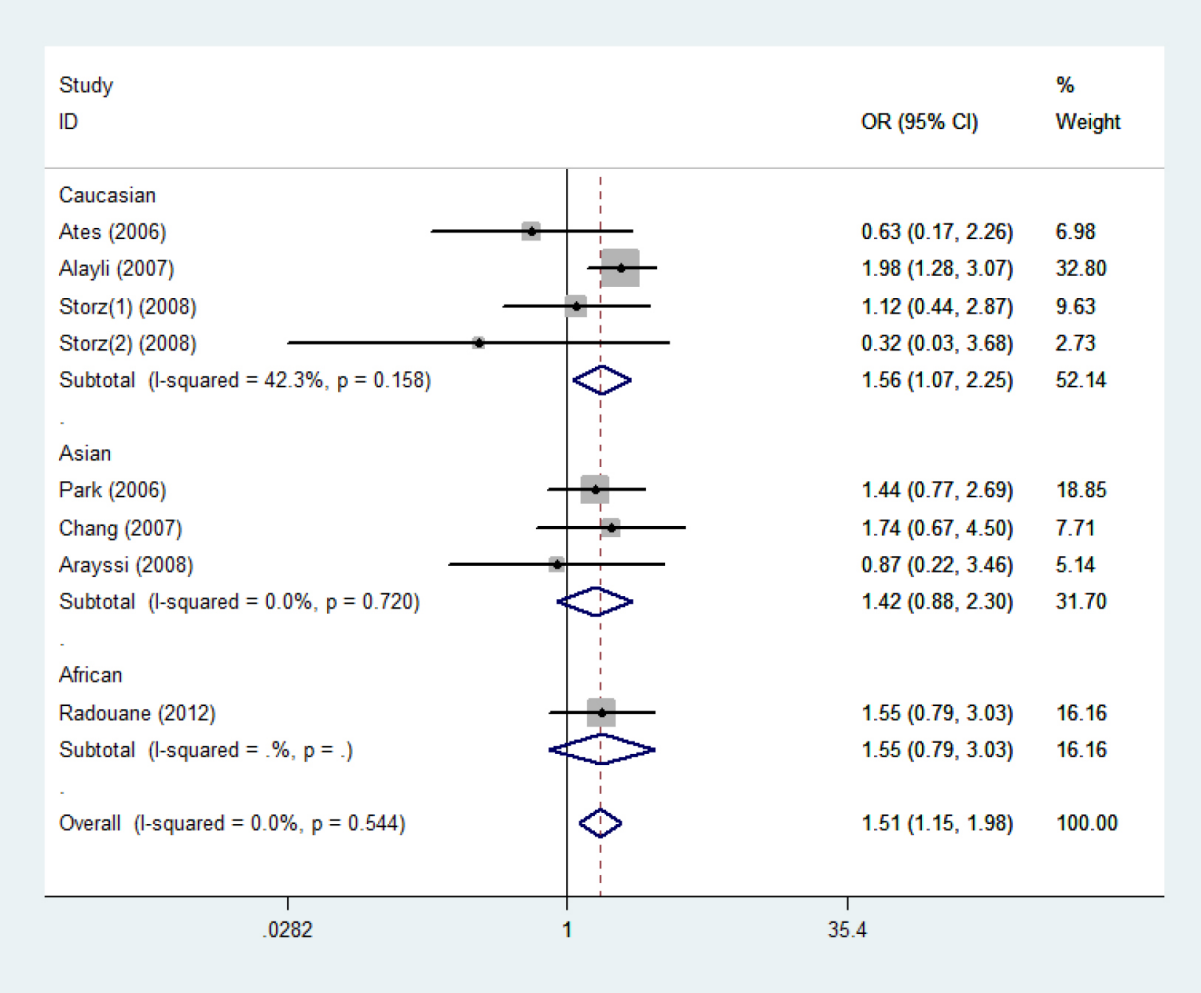
Odds ratios and 95% confidence intervals for individual studies and pooled data for the association between the A versus G allele of the tumor necrosis factor −238A/G polymorphism and Behcet’s disease.

Eight case-control studies including 842 cases and 938 controls identified an association between the TNF −238A/G polymorphism and BD risk [35-38,40,42,47]. The pooled OR (95% CI, p value) in the A versus G allele was 1.512 (1.155–1.979, p=0.003). In the subgroup analysis by ethnicity, we found that the BD cases had a significant higher frequency of A versus G (OR=1.556, 95% CI=1.074–2.253, p=0.019) than that in the controls in the Caucasian populations. The forest plot is shown in Figure 3.

### Tumor necrosis factor −1031C/T polymorphism and Behcet’s disease

Seven studies containing 738 cases and 964 controls examined the association of TNF −1031C/T and BD [23,36,37,39,41,42,44]. Results indicated a significant association between the TNF −1031C/T polymorphism and BD (OR=1.549, 95% CI=1.190–2.015, p=0.001). Stratifying by ethnicity, we found a significant association in the Caucasian population (OR=2.171, 95% CI=1.581–2.981, p<0.001).

### Tumor necrosis factor −857T/C, −863A/C, and −376A/G polymorphisms and Behcet’s disease

Four studies focused on the association between the TNF −857T/C polymorphism and BD risk [36,37,42,47]. The total sample size for patients with BD and healthy controls was 533 and 660, respectively. A significant association was observed in the T versus C allele (OR=0.758, 95% CI=0.593–0.968, p=0.027). Ethnicity-specific analysis showed the −857T allele was significantly associated with BD in the Asian subjects (OR=0.757, 95% CI=0.583–0.983, p=0.037). For two other SNPs, results from the meta-analysis showed that the TNF −863A/C and −376A/G polymorphisms were not susceptible to BD. Detailed results are presented in Table 2.

### Heterogeneity and publication bias

Heterogeneity of the included studies regarding each polymorphism is presented in Table 2. Heterogeneity was found between the TNF −1031C/T and −863A/C polymorphisms and overall BD susceptibility (χ^2^=13.54, I^2^=55.7%, p=0.035; χ^2^=6.31, I^2^=68.3%, p=0.043, respectively). For the TNF −863A/C polymorphism, after stratifying the analyses by ethnicity, we detected significant heterogeneity in the Asian populations (χ^2^=6.15, I^2^=83.7%, p=0.013). Evidence of publication bias was observed for the meta-analysis of the TNF −238A/G in all study subjects and the Caucasian group with a p value for Egger’s linear regression test: 0.002 and 0.020. Thus, the “trim and fill” method was used to adjust for publication bias. The adjusted OR calculation using the “trim and fill” technique remained significant (OR=1.521, 95% CI=1.159–1.995; OR=1.574, 95% CI=1.083–2.288, respectively), suggesting that these results might not be affected by publication bias.

## Discussion

Since the clear pathogenesis of BD remains to be elucidated, it is highly suggestive that multiple host genetic factors are involved in the development of BD [18]. TNF-α is a multifunctional cytokine secreted by monocytes that plays a central role in initiating and regulating the immune response [52]. Recently, genetic variants of the TNF gene have drawn increasing interest in the etiology of several autoimmune diseases [53,54]. Several studies have shown an association of TNF gene polymorphisms in patients with BD, but the results of individual studies were inconsistent. Radouane et al. observed that TNF −1031C constitutes a susceptibility allele for BD and genital ulcers, and reported a strong association between the −238A allele and the absence of uveitis, indicating that the −238A allele could be a good prognostic factor for anterior uveitis [47]. In contrast, Chang et al. discovered no significant difference in the allele frequency of TNF −1031C/T between patients with BD and controls in a Korean population, and the analysis of the influences of the TNF gene on various clinical manifestations of BD showed that TNF −1031C was not related to the presence of clinical features, such as oral and genital ulceration and uveitis [37]. To comprehensively analyze these associations between TNF gene polymorphisms and BD susceptibility, a meta-analysis was performed.

Overall, to our knowledge, this is the first study to confirm the association between the TNF −308A/G polymorphism and BD susceptibility. Significant associations were also identified between the TNF −238A/G, −1031C/T, and −857T/C polymorphisms and BD risk, whereas the TNF −863A/C and −376A/G polymorphisms did not appear to have a significant association with overall BD risk. These results were similar to those observed by Touma et al. in the previous meta-analysis [49].

The findings of the present study seem to contradict individual studies included in the meta-analysis, which are non-significant studies. In this meta-analysis, we found significant differences after pooling all individual studies. The reasons for this disagreement may arise from two aspects. On the one hand, although some studies are non-significant, the ORs (95% CIs) of the individual studies [34,36,37,44,46,47] draw near critical values as shown in Figure 2 and Figure 3. If these individual studies increased the sample size, they might yield significant association. On the other hand, meta-analysis is a means of increasing the effective sample size under investigation through pooling data from individual association studies, and can overcome the limitations of individual studies, resolve inconsistencies, and reduce the likelihood that random errors are responsible for false-positive or false-negative associations; therefore, meta-analysis can enhance the statistical power of the analysis for estimating genetic effects.

In the present study, we also preformed subgroup analyses by ethnicity for these polymorphisms. Our results revealed that the −308A/G and −857T/C polymorphisms were associated with BD only in Asians, while the −238A/G and −1031C/T polymorphisms were associated with BD in Caucasians. The meta-analysis of the −1031C/T polymorphism showed a significant association with Africans, but it might not be reliable because only two published articles in African population were included in the present study. Therefore, additional large sample size case-control studies should be performed in this group.

The diverse roles of the same gene polymorphism in subgroup analysis by ethnicity could be ascribed to the following major aspects. First, BD is a complex autoimmune disease, and genetic heterogeneity exists in different populations. GWASs on BD have confirmed this genetic heterogeneity [9,10]. Similarly, rheumatoid arthritis is also a complex autoimmune disease, and genetic heterogeneity exists in different populations. GWASs have determined genetic heterogeneity for TRAF1/C5 [55,56]. Second, autoimmune diseases are multifactorial and caused by an interaction of genetic and environmental factors. Gene-environment interactions of different populations are not all the same, and are partly affected by the various environment backgrounds, which may often play a different role in autoimmune diseases susceptibility [13,14]. Genetic and environment factors play a key role in disease initiation of systemic lupus erythematosus as well as its evolution. A previous study demonstrated that TNF −238A/G was associated with systemic lupus erythematosus in Caucasian populations, not in African and Mexican populations, suggesting the interactions between different environments and gene might be different [57]. Third, different linkage disequilibrium (LD) patterns may contribute to the discrepancy. The TNF gene is located at the class III region of the HLA complex, adjacent to HLA-B [32], and the MHC/HLA complex is the most polymorphic genetic region [58,59]. A polymorphism may be in LD with a nearby causal variant in one ethnic group, but not in another.

Compared with the previous meta-analysis [49], the current study involved a total of 16 articles, which is larger than the data from the previous meta-analysis. Moreover, we performed subgroup analyses by ethnicity to look at the ethnic effect on the risk of BD. In addition, several studies have reported significant associations between genetic polymorphisms and diseases when the genotype distribution of the control population deviated from HWE, but deviation from HWE in the control population might imply potential selection biases of controls or genotype errors. Therefore, we excluded studies in which HWE was absent in the controls. Thus, our meta-analysis might draw a more reliable conclusion.

Some limitations of the present study should be considered. First, this study could not analyze the potential gene-environment interactions and gene susceptibility haplotypes owing to lack of data, such as data on environmental risk factors and genotypes. Second, ocular involvement is frequent and severe, but this study could not assess the association between TNF gene polymorphisms and ocular inflammation because of the insufficient data. Third, our literature search was dependent on English and Chinese; language bias might be considered. Fourth, although adjustment using the “trim and fill” method did not affect the results of the meta-analysis, publication bias still existed, and it might have influenced the current meta-analysis. Finally, different genotyping methods and disease status might affect the data interpretation of the included studies.

In summary, this updated meta-analysis suggests that TNF −308G, −238A, −1031C, and −857C alleles might be risk alleles for BD susceptibility. However, a large sample size including more ethnic groups with careful matching between cases and controls should be considered in future association studies to confirm the results of our meta-analysis.
